# Supplementary material for: Assessing drug safety by identifying the axis of arrhythmia in cardiomyocyte electrophysiology
Source: eLife. 2023 Dec 11;12:RP90027. doi: 10.7554/eLife.90027 (PMC10712948; doi:10.7554/eLife.90027)
Supplement: Table 2—source data 2. [file elife-90027-table2-data2.pdf]

Table 2 – source data 2

Drugs with Class 1 Torsadogenic risk

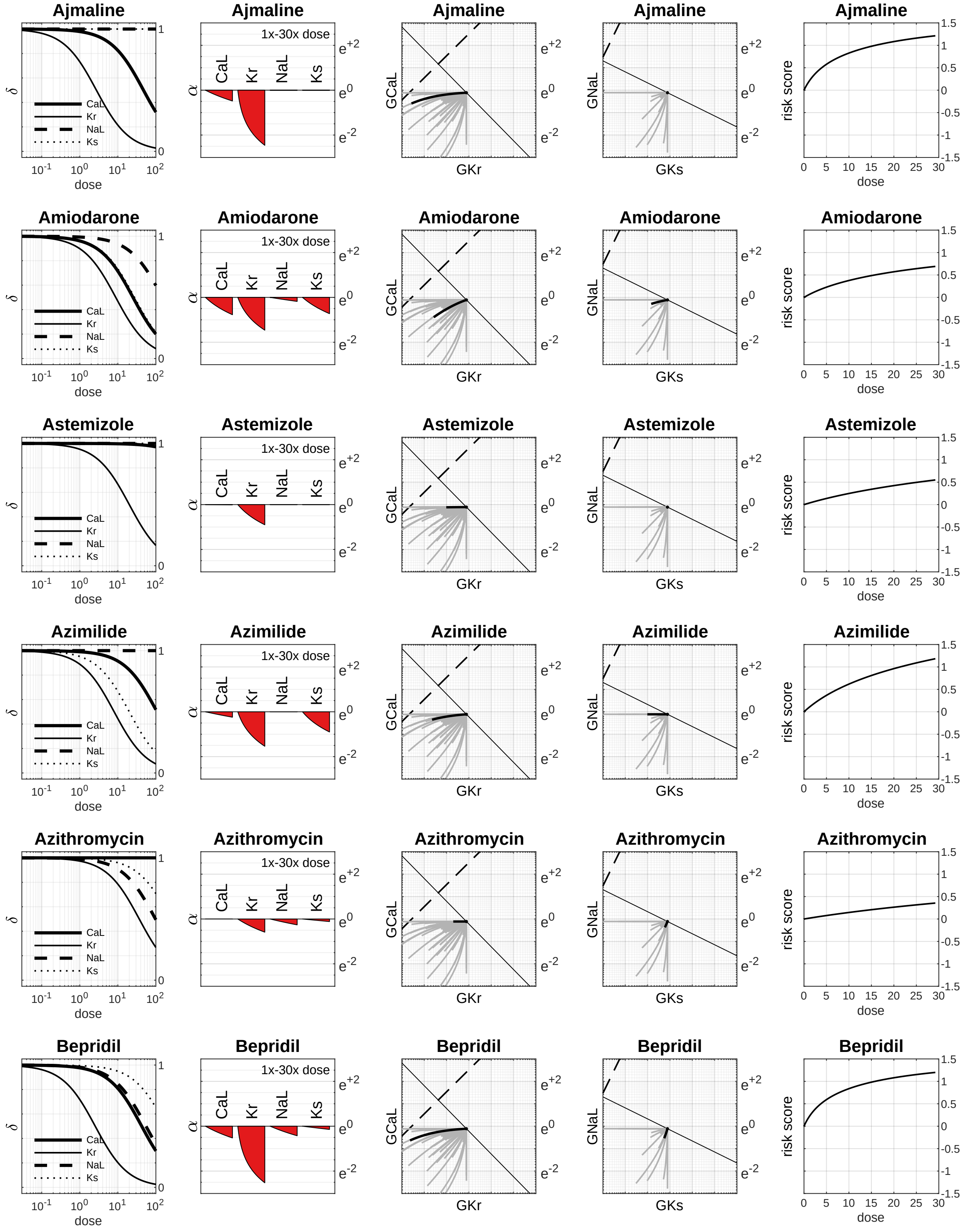

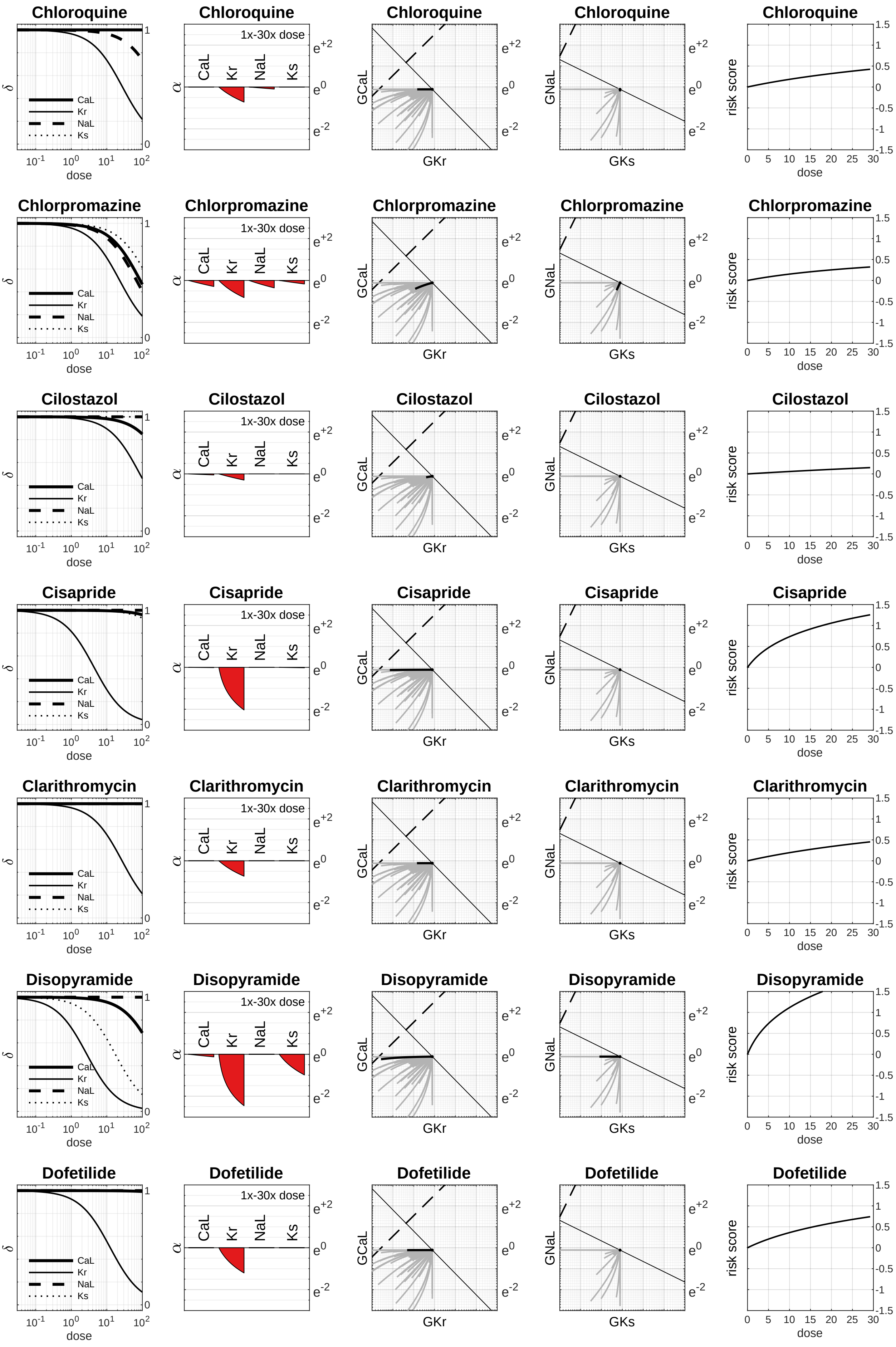
Chloroquine
Chlorpromazine
Cilostazol
Cisapride
Clarithromycin
Disopyramide
Dofetilide
δ
dose
10^{-1}
10^{0}
10^{1}
10^{2}
0
1
CaL
Kr
NaL
Ks
α
1x-30x dose
e^{+2}
e^{0}
e^{-2}
GCaL
GKr
GNaL
GKs
risk score
1.5
1
0.5
0
-0.5
-1
-1.5
0
5
10
15
20
25
30

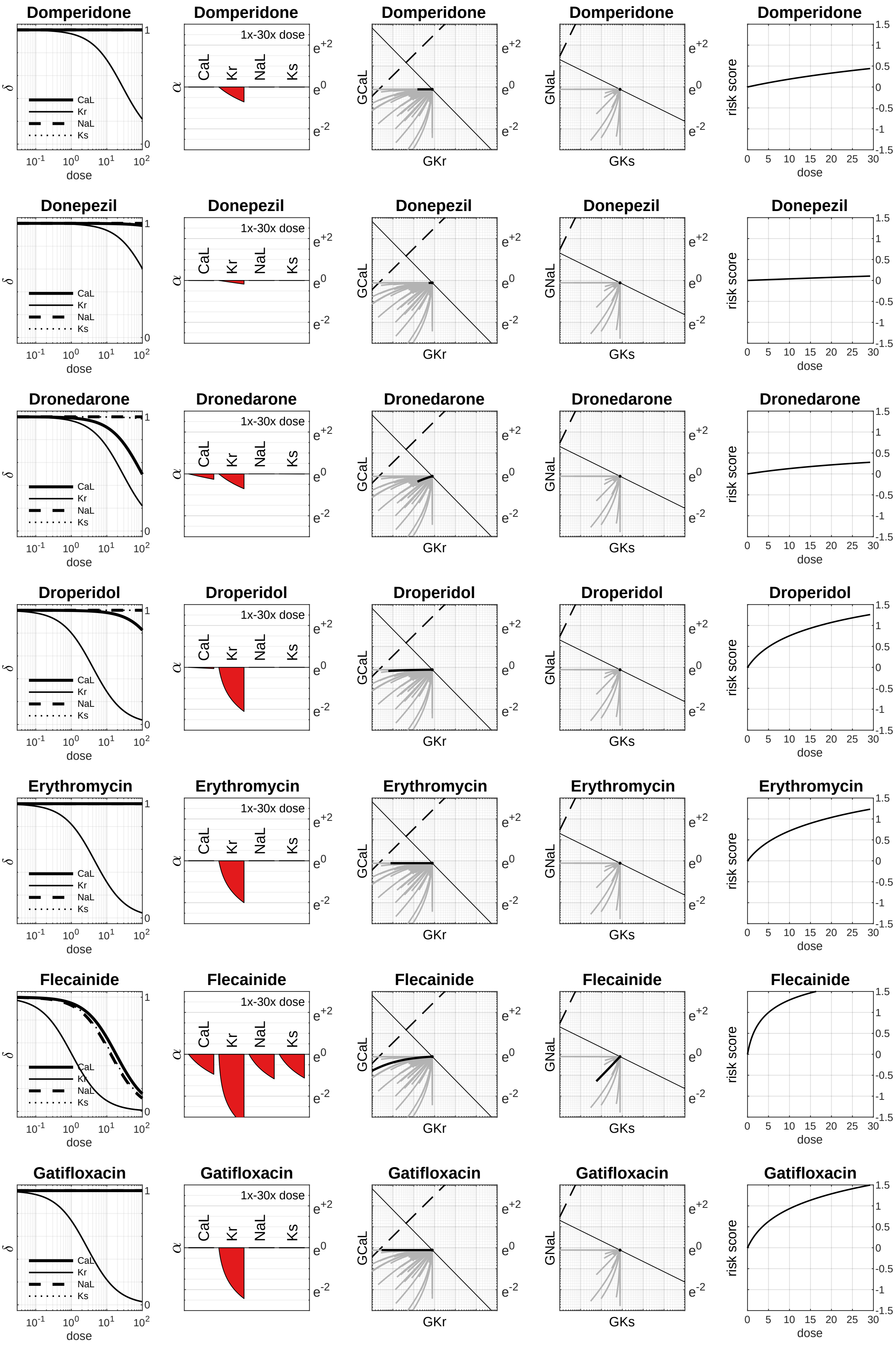
Domperidone
Donepezil
Dronedarone
Droperidol
Erythromycin
Flecainide
Gatifloxacin
δ
dose
CaL
Kr
NaL
Ks
α
1x-30x dose
GCaL
GKr
GNaL
GKs
risk score

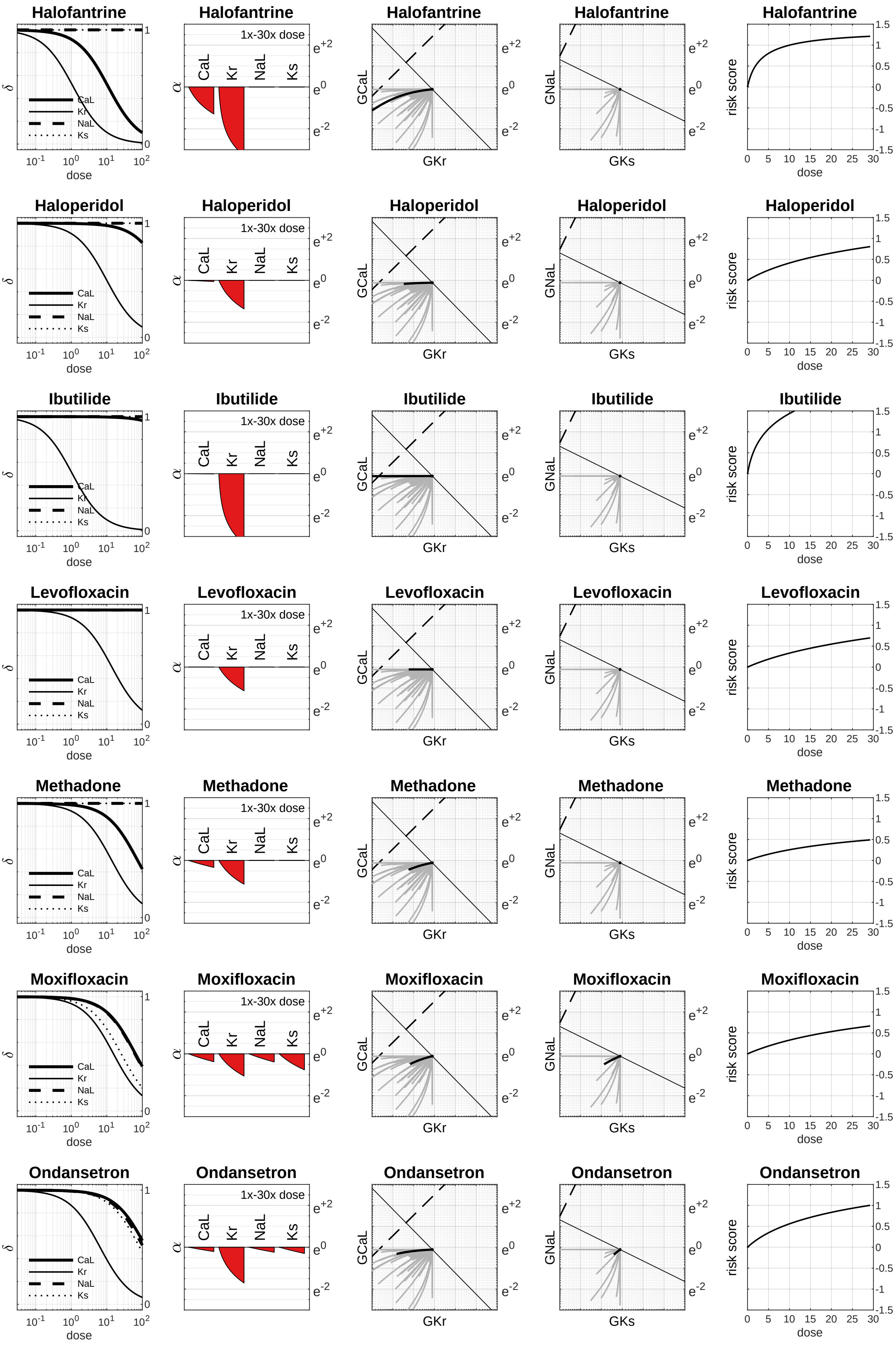
Halofantrine
Haloperidol
Ibutilide
Levofloxacin
Methadone
Moxifloxacin
Ondansetron
δ
dose
CaL
Kr
NaL
Ks
α
1x-30x dose
e+2
e0
e-2
GCaL
GKr
GNaL
GKs
risk score

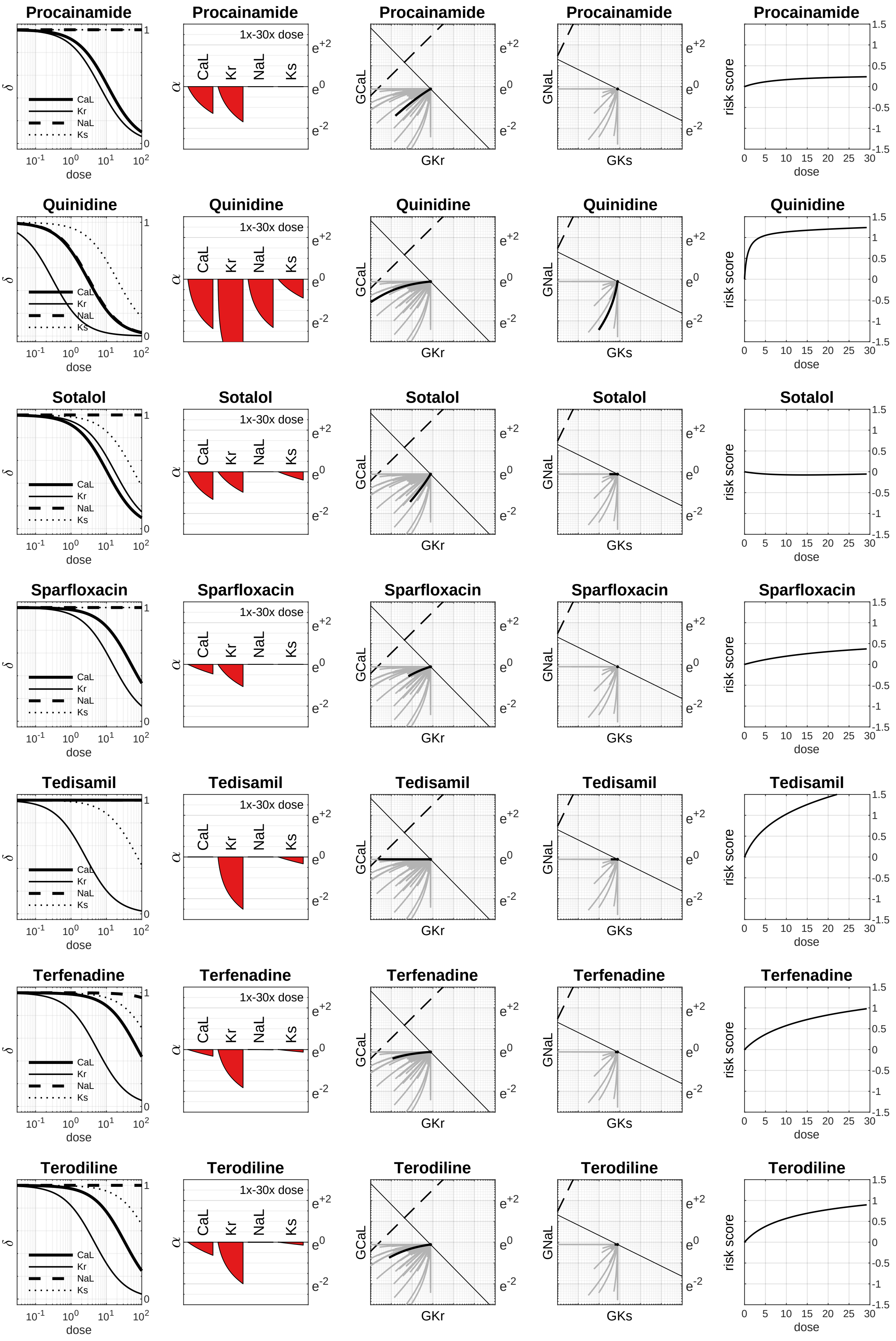
Procainamide
Quinidine
Sotalol
Sparfloxacin
Tedisamil
Terfenadine
Terodiline
δ
dose
CaL
Kr
NaL
Ks
α
1x-30x dose
e+2
e0
e-2
GCaL
GKr
GNaL
GKs
risk score

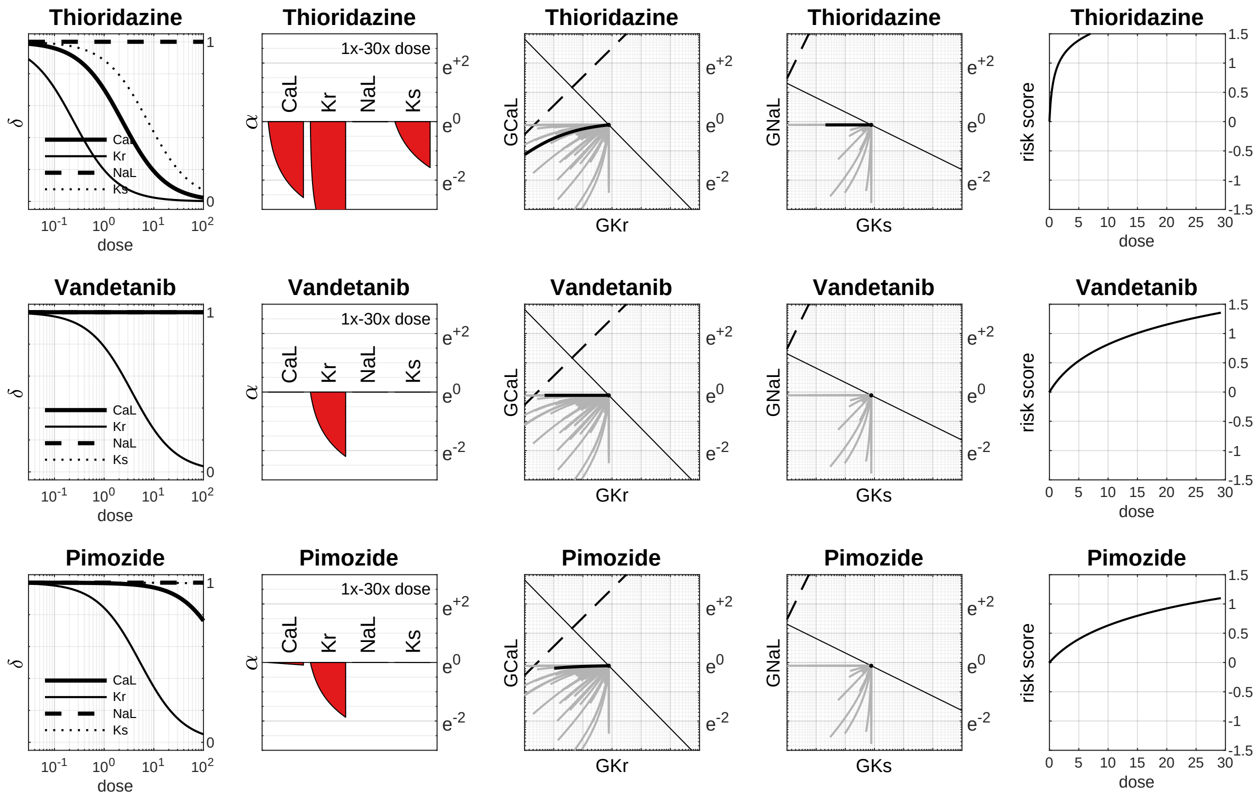

Drugs with Class 2 Torsadogenic risk

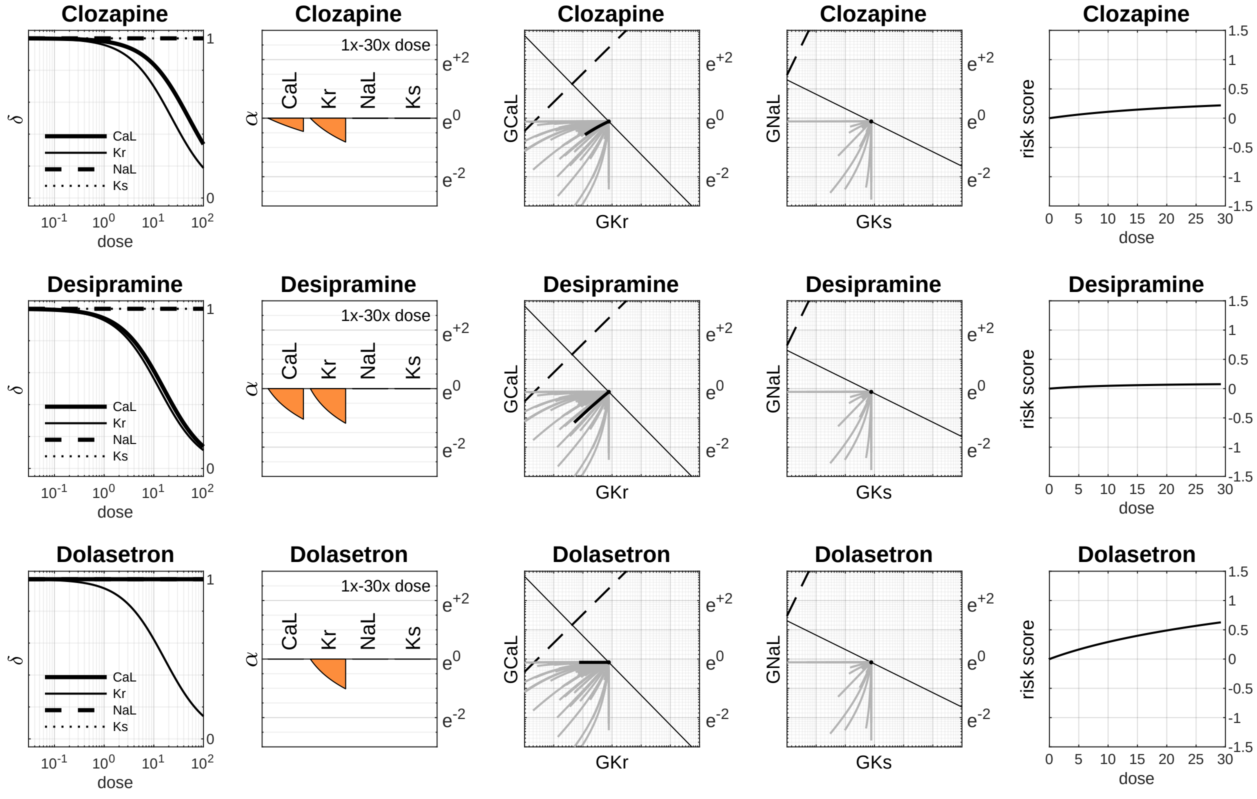

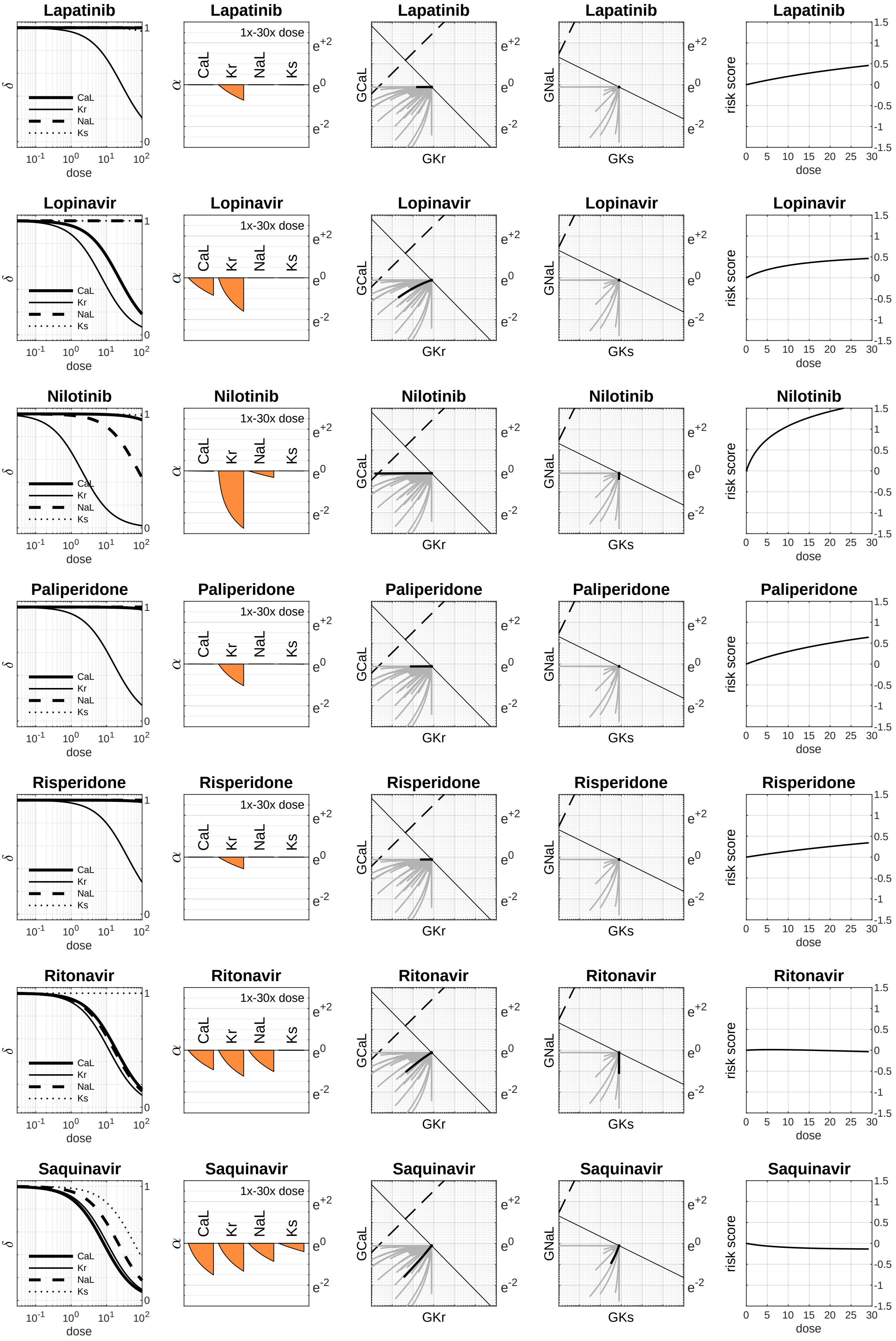
Lapatinib
Lopinavir
Nilotinib
Paliperidone
Risperidone
Ritonavir
Saquinavir
δ
dose
CaL
Kr
NaL
Ks
α
1x-30x dose
GCaL
GKr
GNaL
GKs
risk score

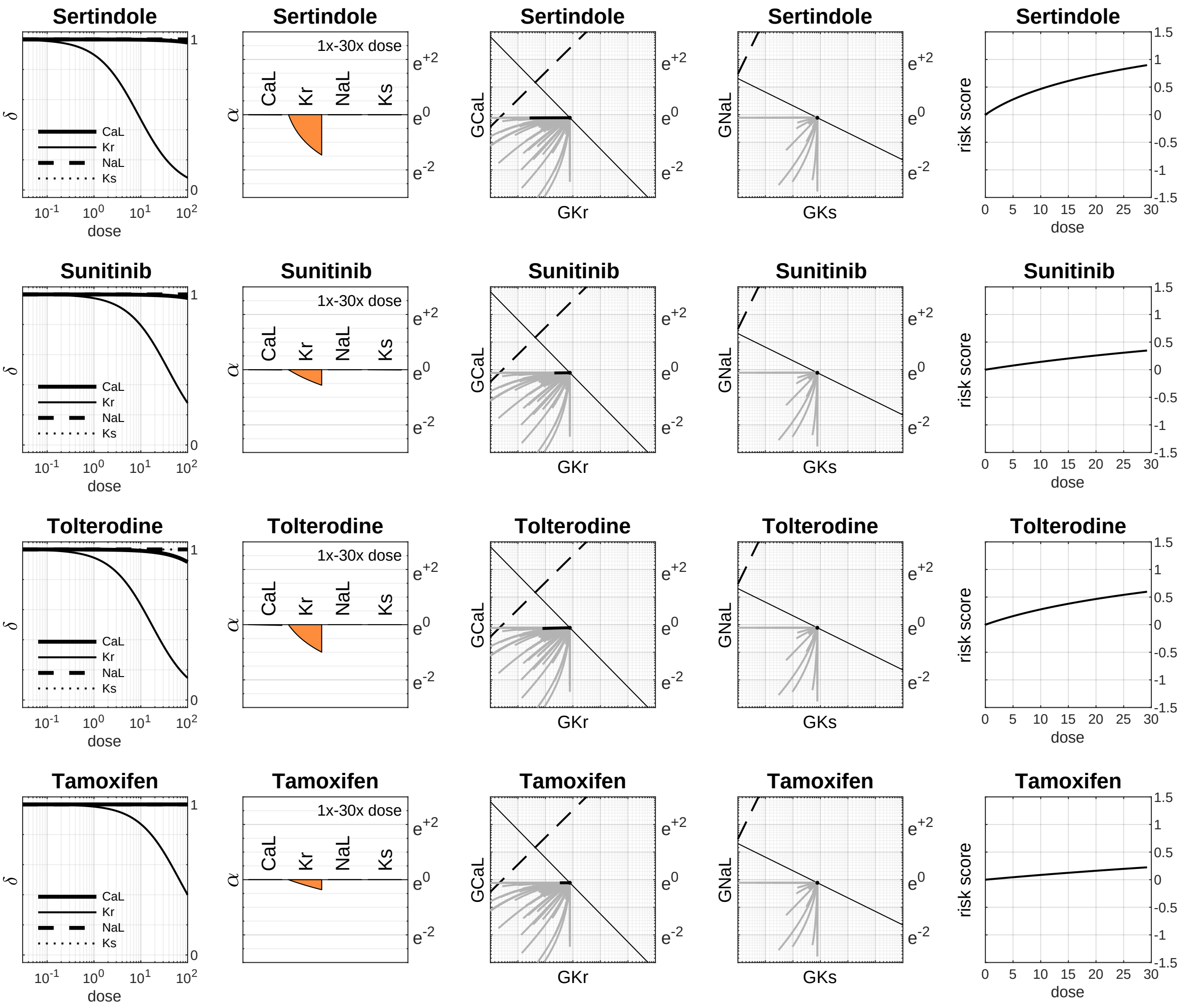

Drugs with Class 3 Torsadogenic risk

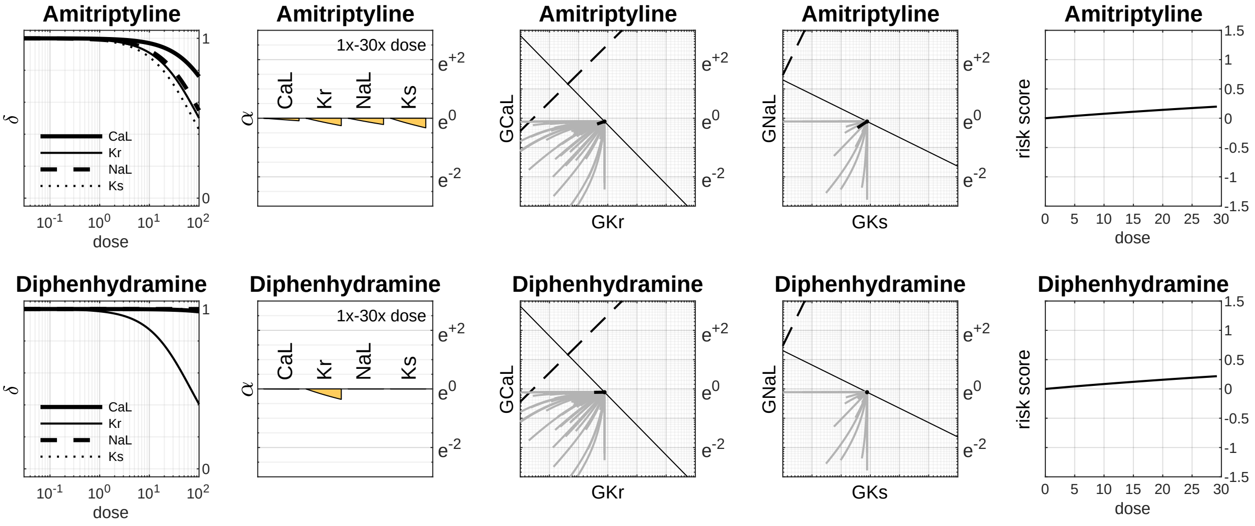

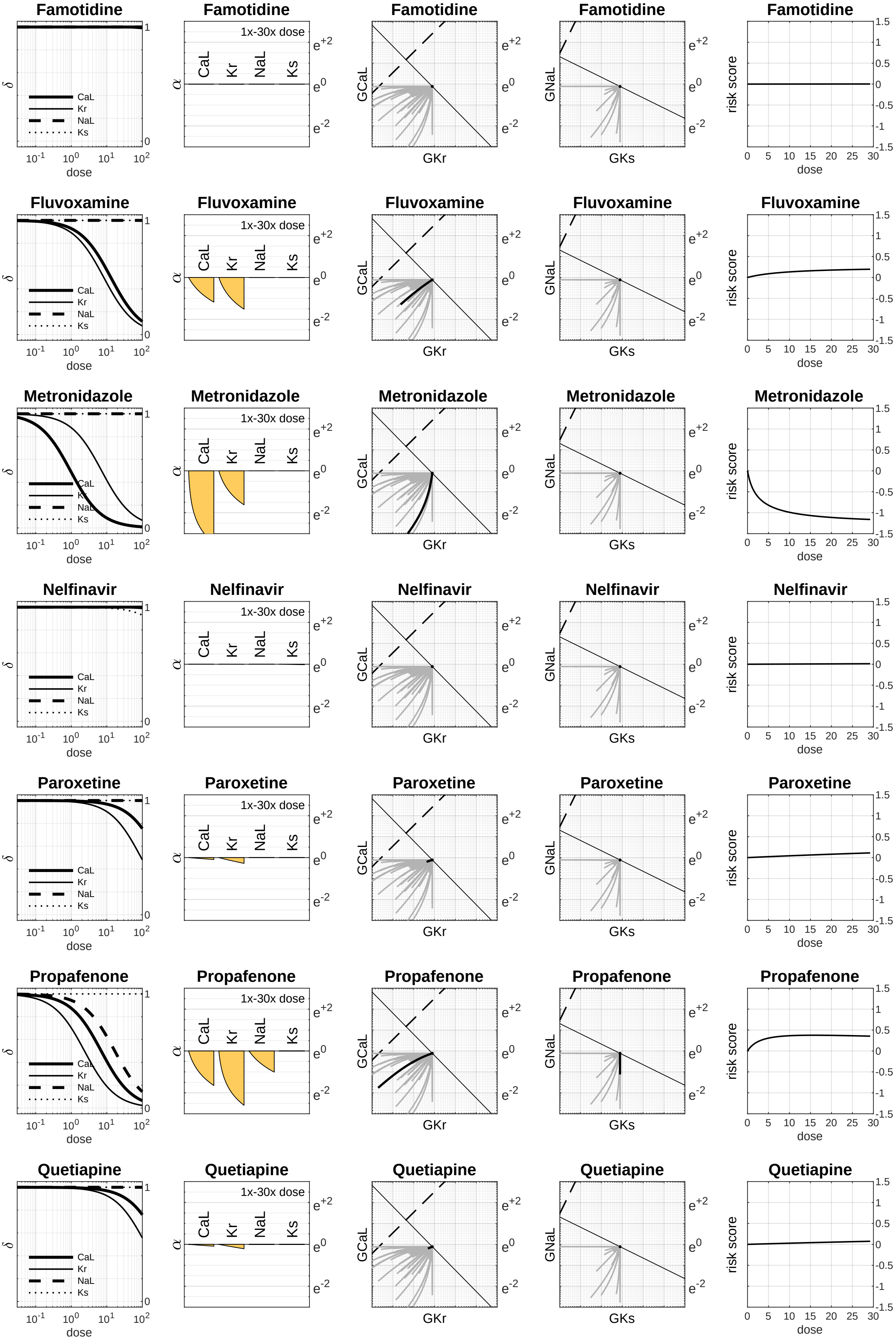
Famotidine
Fluvoxamine
Metronidazole
Nelfinavir
Paroxetine
Propafenone
Quetiapine
δ
dose
CaL
Kr
NaL
Ks
α
1x-30x dose
e^{+2}
e^{0}
e^{-2}
GCaL
GKr
GNaL
GKs
risk score
1.5
1
0.5
0
-0.5
-1
-1.5
0
5
10
15
20
25
30
10^{-1}
10^{0}
10^{1}
10^{2}

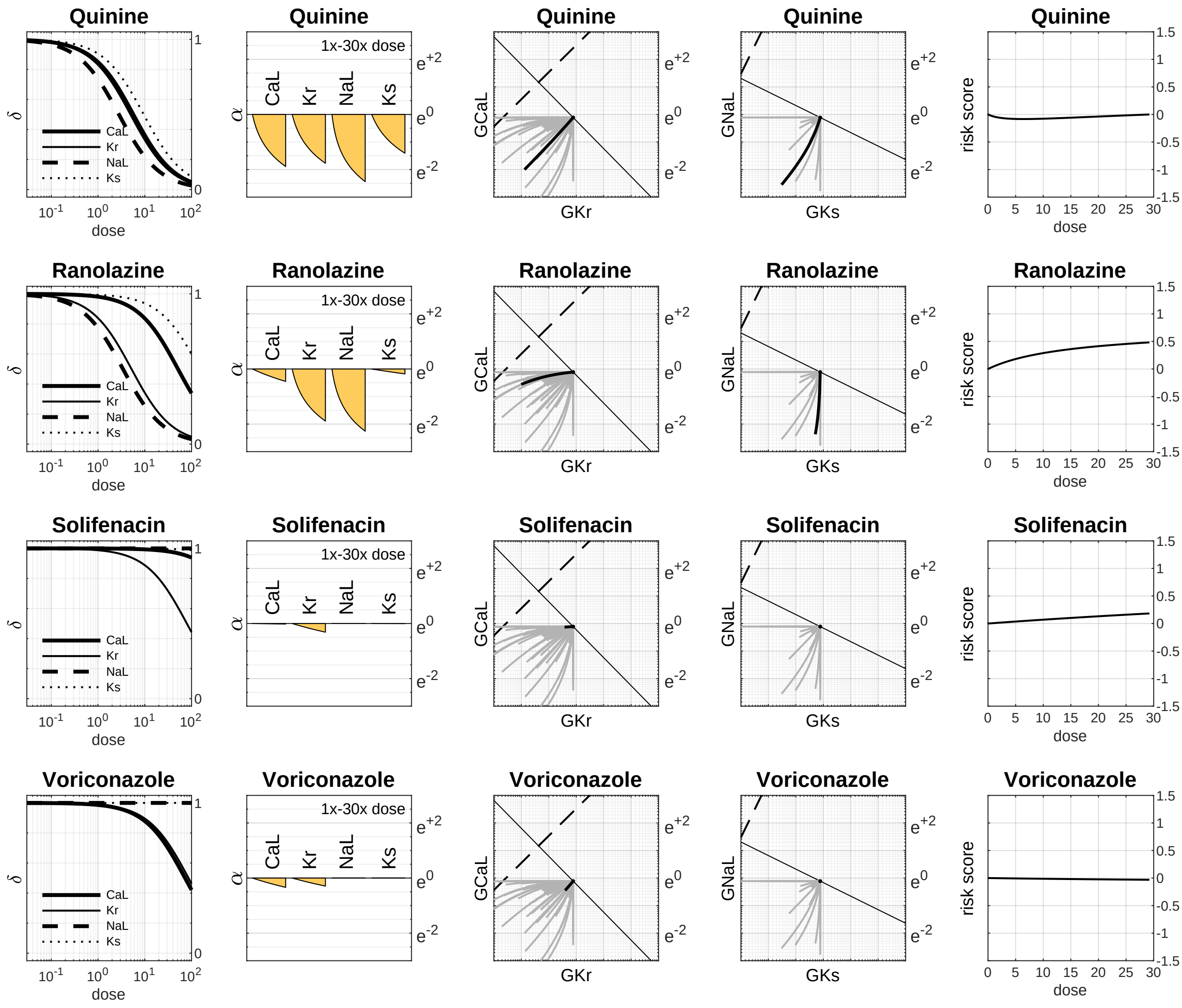

Drugs with Class 4 Torsadogenic risk

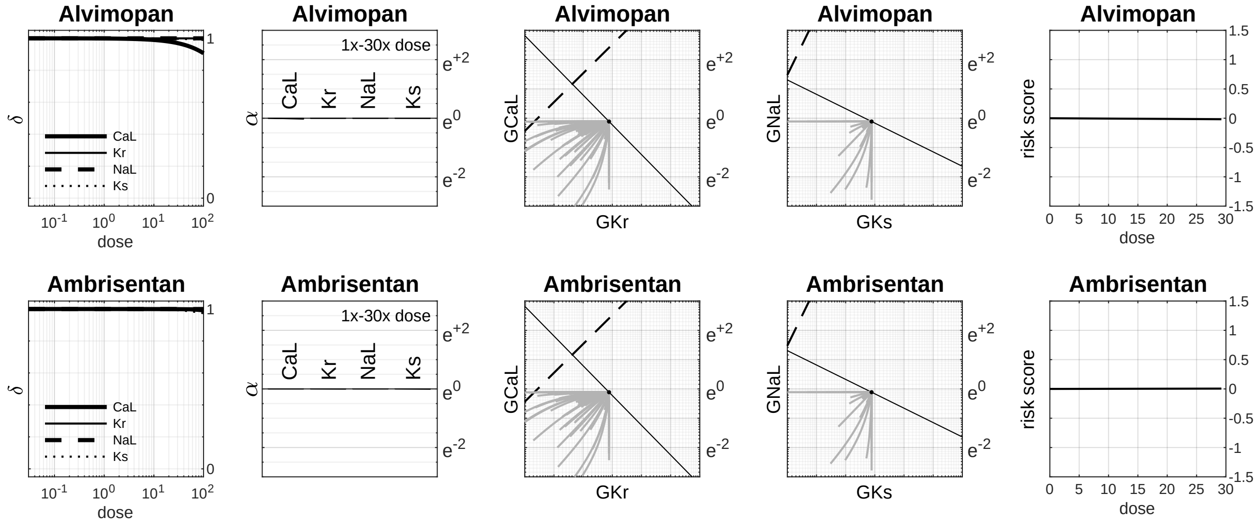

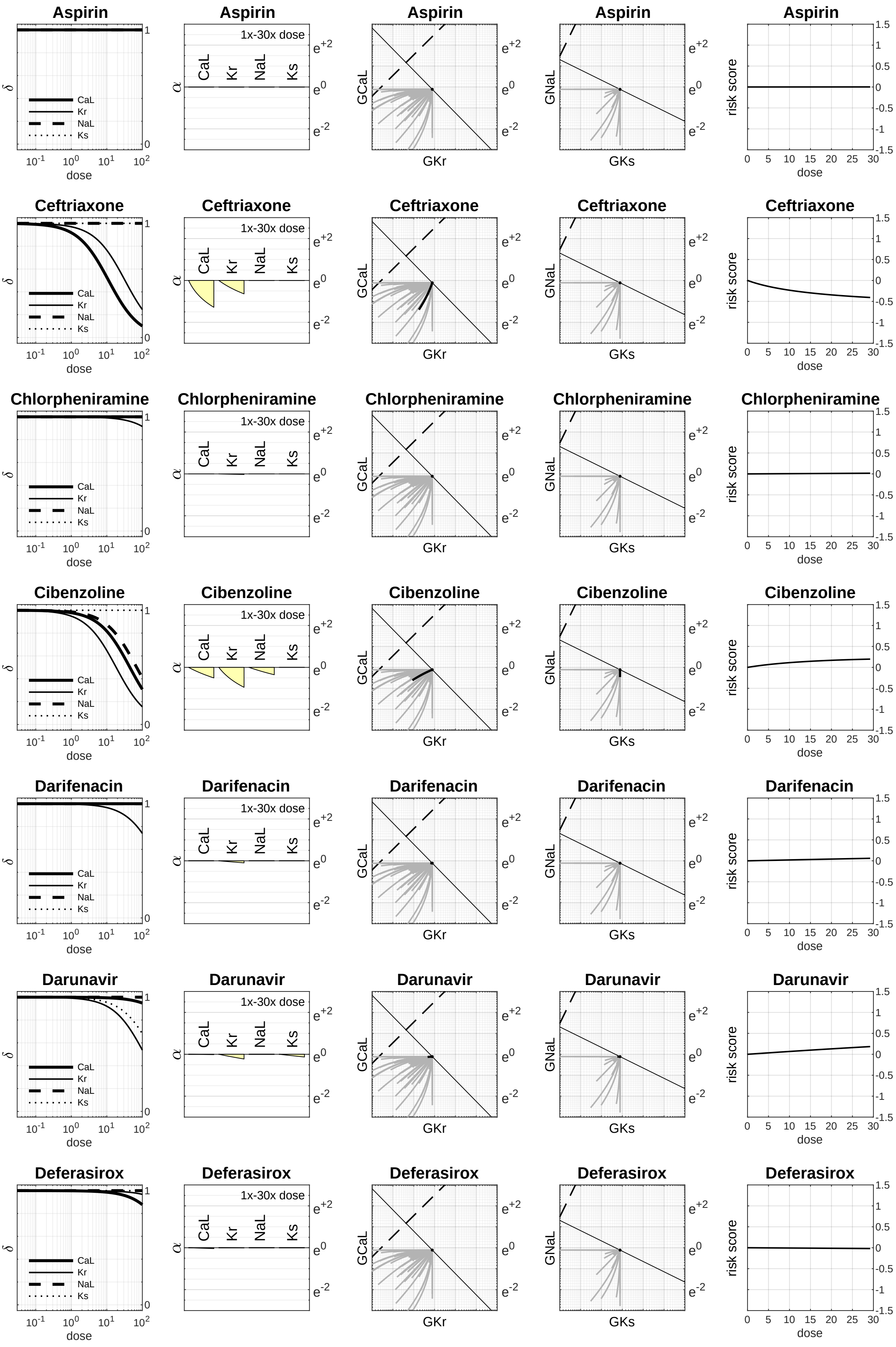

Aspirin
Ceftriaxone
Chlorpheniramine
Cibenzoline
Darifenacin
Darunavir
Deferasirox
δ
dose
CaL
Kr
NaL
Ks
α
1x-30x dose
GCaL
GKr
GNaL
GKs
risk score

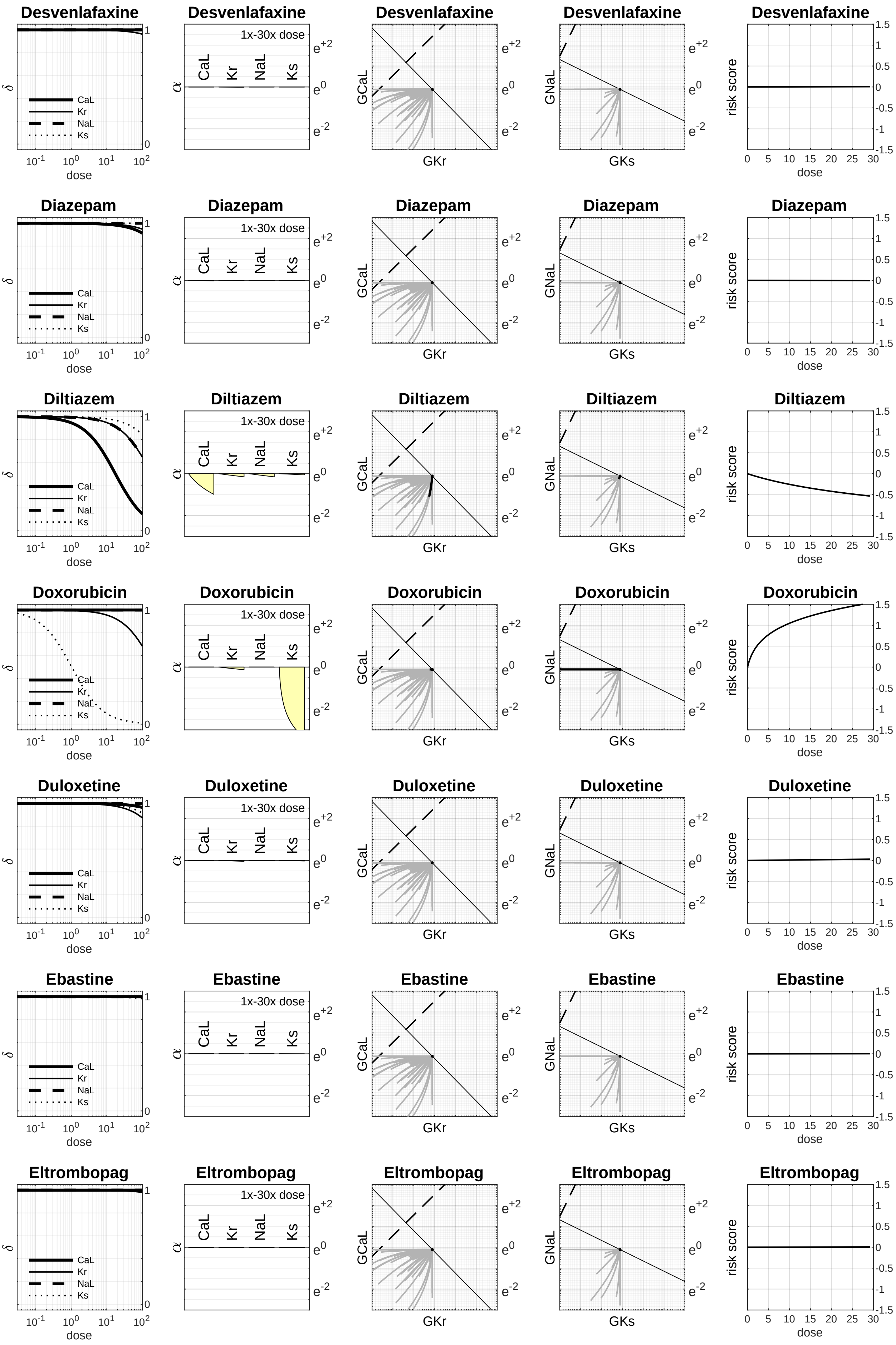
Desvenlafaxine
Diazepam
Diltiazem
Doxorubicin
Duloxetine
Ebastine
Eltrombopag
δ
dose
CaL
Kr
NaL
Ks
1x-30x dose
α
GCaL
GKr
GNaL
GKs
risk score

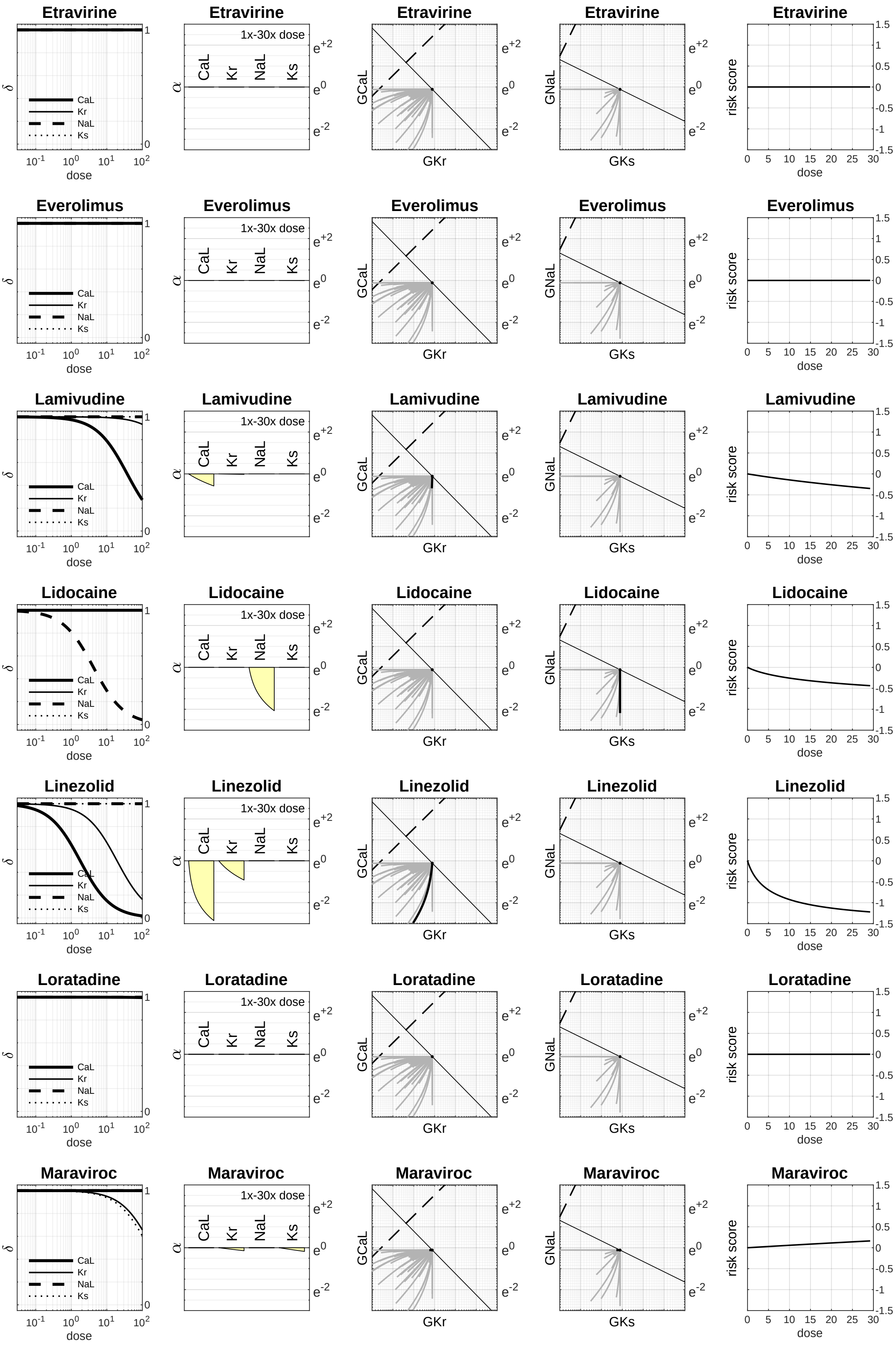

Etravirine
Everolimus
Lamivudine
Lidocaine
Linezolid
Loratadine
Maraviroc
δ
dose
10^{-1} 10^{0} 10^{1} 10^{2}
1
0
CaL
Kr
NaL
Ks
α
1x-30x dose
CaL
Kr
NaL
Ks
e^{+2}
e^{0}
e^{-2}
GCaL
GKr
GNaL
GKs
risk score
1.5
1
0.5
0
-0.5
-1
-1.5
0 5 10 15 20 25 30

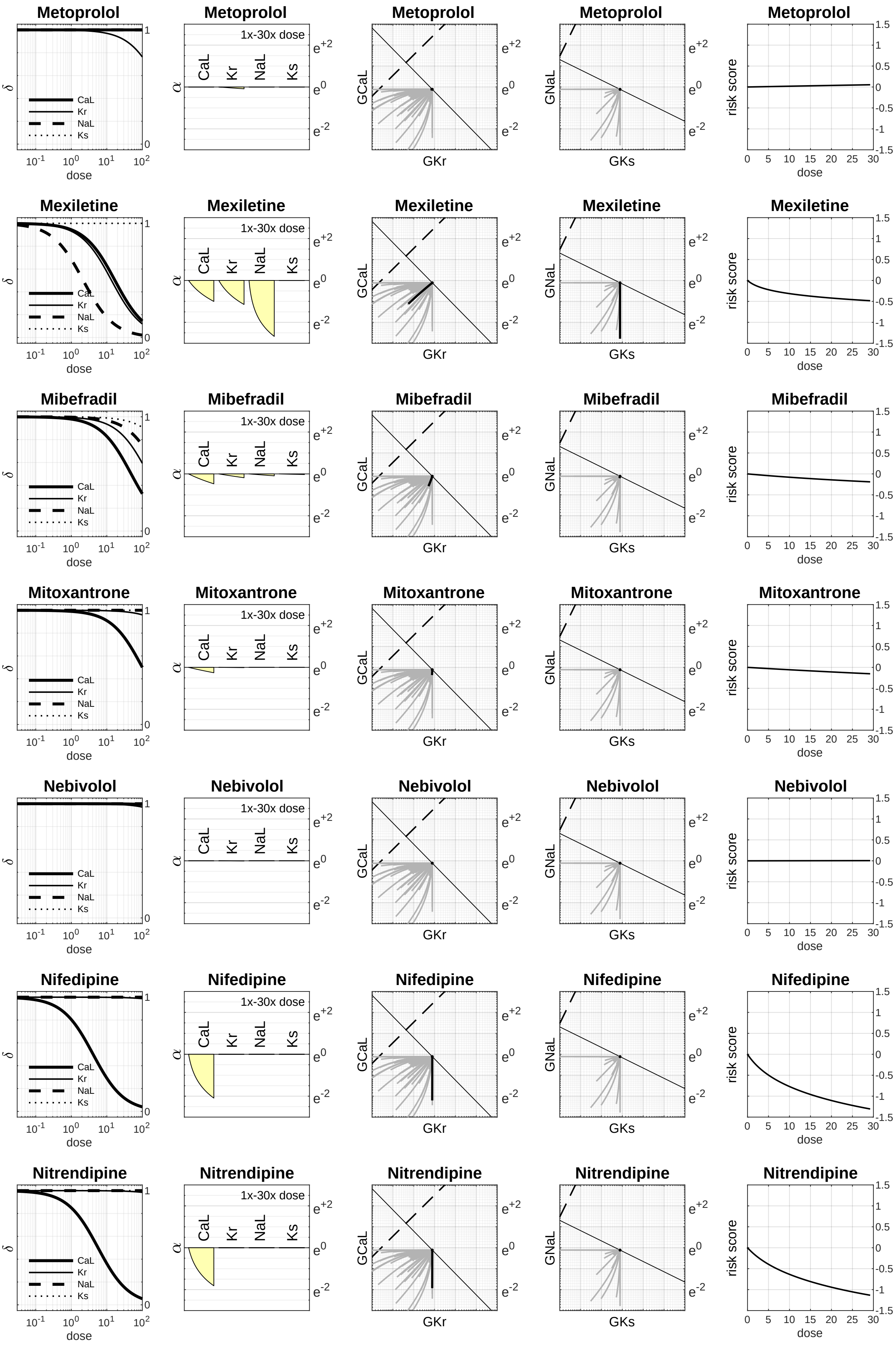
Metoprolol
Mexiletine
Mibefradil
Mitoxantrone
Nebivolol
Nifedipine
Nitrendipine
δ
dose
10^{-1} 10^{0} 10^{1} 10^{2}
CaL
Kr
NaL
Ks
α
1x-30x dose
e^{+2}
e^{0}
e^{-2}
GCaL
GKr
GNaL
GKs
risk score
1.5 1 0.5 0 -0.5 -1 -1.5
0 5 10 15 20 25 30

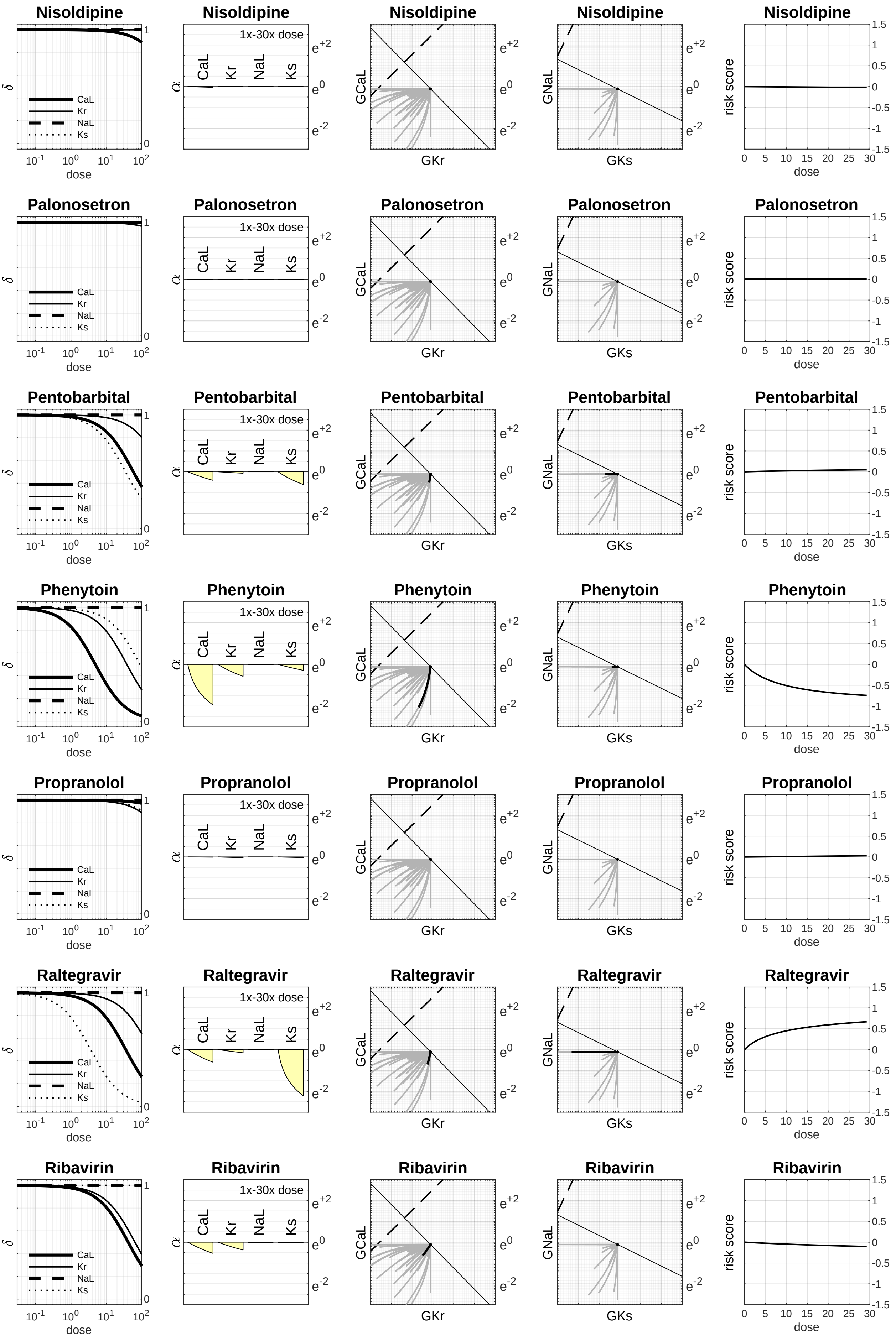

Nisoldipine
Palonosetron
Pentobarbital
Phenytoin
Propranolol
Raltegravir
Ribavirin
δ
dose
CaL
Kr
NaL
Ks
α
1x-30x dose
GCaL
GKr
GNaL
GKs
risk score

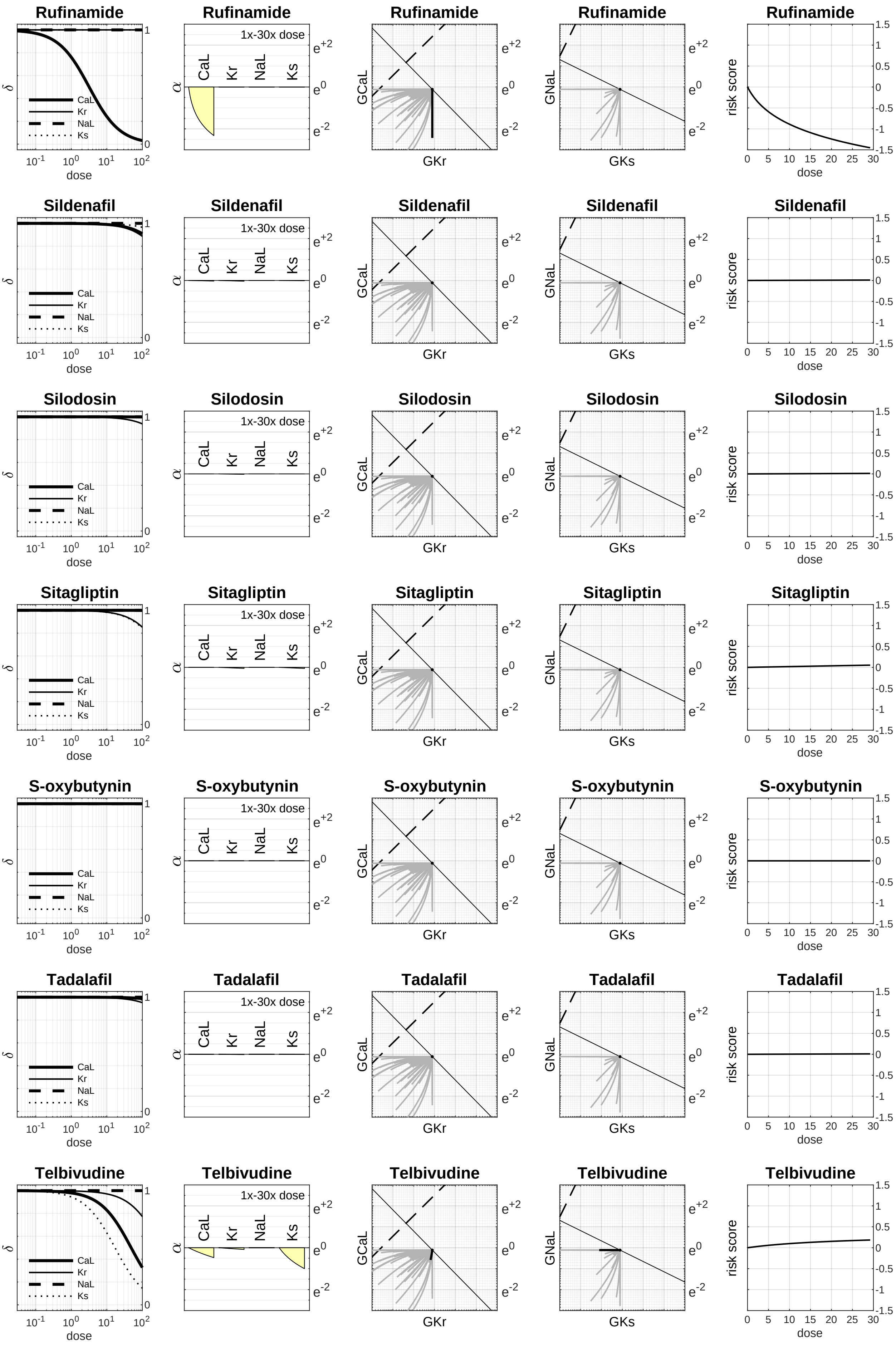
Rufinamide
Sildenafil
Silodosin
Sitagliptin
S-oxybutynin
Tadalafil
Telbivudine
δ
dose
CaL
Kr
NaL
Ks
1x-30x dose
α
GCaL
GKr
GNaL
GKs
risk score

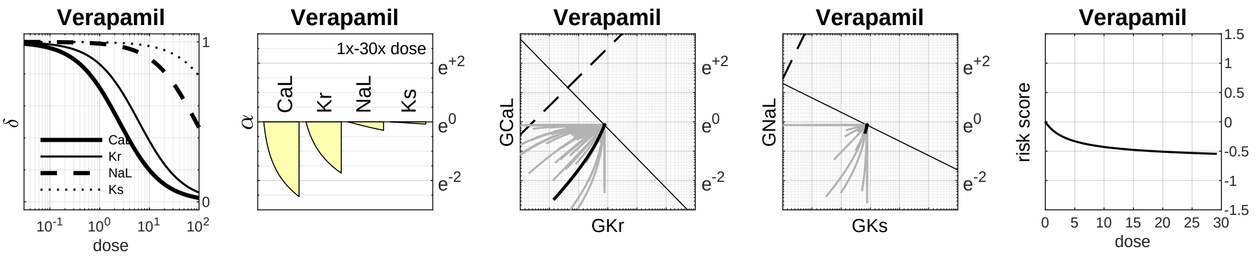
Verapamil
δ
dose
CaL
Kr
NaL
Ks
1x-30x dose
α
GCaL
GKr
GNaL
GKs
risk score
e+2
e0
e-2
